# Patient-Derived Extracellular Vesicles Proteins as New Biomarkers in Multiple Myeloma - A Real-World Study

**DOI:** 10.3389/fonc.2022.860849

**Published:** 2022-06-21

**Authors:** Bruna Velosa Ferreira, Emilie Arnault Carneiro, Carolina Pestana, Filipa Barahona, Joana Caetano, Raquel Lopes, Paulo Lúcio, Manuel Neves, Hans Christian Beck, Ana Sofia Carvalho, Rune Matthiesen, Bruno Costa-Silva, Cristina João

**Affiliations:** ^1^ Myeloma Lymphoma Research Group, Champalimaud Experimental Clinical Research Programme, Champalimaud Foundation, Lisbon, Portugal; ^2^ NOVA Medical School (NMS), NOVA University Lisbon, Lisbon, Portugal; ^3^ Hemato-Oncology Unit, Champalimaud Clinical Centre, Champalimaud Foundation, Lisbon, Portugal; ^4^ Centre of Statistics and its Applications, Faculty of Sciences, University of Lisbon, Lisbon, Portugal; ^5^ Faculty of Medicine, University of Lisbon, Lisbon, Portugal; ^6^ Centre for Clinical Proteomics, Clinical Biochemistry and Pharmacology, Odense University Hospital, Odense, Denmark; ^7^ NOVA Medical School (NMS), Faculdade de Ciências Médicas (FCM), Universidade Nova de Lisboa, Lisboa, Portugal; ^8^ Systems Oncology Group, Champalimaud Physiology and Cancer Programme, Champalimaud Foundation, Lisbon, Portugal

**Keywords:** extracellular vesicles (EV), multiple myeloma, biomarkers, protein, liquid biopsy

## Abstract

Multiple myeloma (MM) is a hematological malignancy of clonal antibody–secreting plasma cells (PCs). MM diagnosis and risk stratification rely on bone marrow (BM) biopsy, an invasive procedure prone to sample bias. Liquid biopsies, such as extracellular vesicles (EV) in peripheral blood (PB), hold promise as new minimally invasive tools. Real-world studies analyzing patient-derived EV proteome are rare. Here, we characterized a small EV protein content from PB and BM samples in a cohort of 102 monoclonal gammopathies patients routinely followed in the clinic and 223 PB and 111 BM samples were included. We investigated whether EV protein and particle concentration could predict an MM patient prognosis. We found that a high EV protein/particle ratio, or EV cargo >0.6 µg/10^8^ particles, is related to poorer survival and immune dysfunction. These results were supported at the protein level by mass spectrometry. We report a set of PB EV-proteins (*PDIA3*, *C4BPA*, *BTN1A1*, and *TNFSF13*) with a new biomarker potential for myeloma patient outcomes. The high proteomic similarity between PB and BM matched pairs supports the use of circulating EV as a counterpart of the BM EV proteome. Overall, we found that the EV protein content is related to patient outcomes, such as survival, immune dysfunction, and possibly treatment response.

## 1 Introduction

MM is a hematological malignancy characterized by the accumulation of clonal plasma cells (PCs) within the bone marrow (BM) and progressive immune dysfunction. The natural progression of myeloma includes the monoclonal gammopathies of uncertain significance (MGUS) and smoldering multiple myeloma (MM) asymptomatic phases, characterized by clonal PCs in the BM without organ damage or a myeloma-defining event (1). Current risk stratification requires BM PC phenotypic/genotypic characterization. Given clonal PC patchy distribution, BM biopsies may not reflect disease heterogeneity (2) while submitting patients to invasive procedures. Finally, scoring systems can be improved as patients within the same prognostic categories may still have very different outcomes. Liquid biopsies may overcome these limitations since they are minimally invasive and less prone to spatial bias and allow for serial sampling. They encompass collecting biofluids (e.g., PB) to obtain a subset of circulating tumor components such as extracellular vesicles (EV), which are bilayer lipid particles naturally released from all cells (3). In recent years, their value as key players in precision medicine has been demonstrated (4). In cancer, EV are emergent actors in intercellular communication transferring cargo molecules (e.g., microRNA and proteins) influencing the phenotype and function of target cells (5). In MM, there is evidence that EV intervenes in key processes such as tumor progression (6), immunosuppression (7), and drug resistance (8). Most studies analyzing MM EV have been focused on the genome, and real-world studies on the EV proteome are scarce. Here, we assessed MM patient EV across routine hemato-oncology clinical practice. Our results demonstrated that the EV protein content is potentially associated to MM patient outcomes such as immune dysfunction, survival, and response to treatment.

## 2 Materials and Methods

**Clinical study:** Healthy donors (HDs), MGUS, and MM patients followed at two hematology departments were included from May 2016 to July 2020, after study approvals. All procedures followed the Helsinki Declaration and were approved by the Institutions’ Ethics Committee, and participants gave written informed consent before inclusion. Disease and response assessments followed the International Myeloma Working Group guidelines (9). Patients’ PB and/or BM samples were collected prospectively according to the clinical follow-up, meaning that MM patients were included before treatment and/or at disease evaluation (all lines). The MM status at the sample collection was defined as smoldering (SMM), newly diagnosed (MM-ND), responders (MM-R) if in partial response or better, or non-responders (MM-NR) if a stable or progressive disease. HDs only collected PB. The patient and sample distribution for each analysis is described in **Figure S1**. Overall survival (OS) refers to the time from inclusion to death by any cause.

**Small EV purification:** PB and BM samples were centrifuged at 500 g for 10 min. The collected supernatant was centrifuged at 3,000 g for 20 min at 4°C, and plasma was stored at -80°C. Sequential ultracentrifugation with gradient density was performed as previously described (10), permitting intermediate EV recovery with intermediate specificity. Detailed protocols can be found in supplementary material.

**Transmission electron-microscopy (TEM):** Purified EV were absorbed onto formvar/carbon-coated glow-discharged copper EM grids (5 μl on each grid) for 20 min; then fixed with 2% formaldehyde, 20min. Grids were stained with 2% uranyl acetate for 5 min. TEM was performed using FEI Tecnai G2 Spirit BioTWIN TEM operating at an accelerating voltage of 120 keV. Images were acquired using an Olympus-SIS Veleta CCD Camera.

**Western blot:** The presence and purity of EV were assessed by western blot using 5ug of protein per sample. CD9 was used as an EV-positive biomarker and APOA1 as a purity biomarker (3).

**EV characteristics:** EV protein concentration was determined by the colorimetric bicinchoninic acid protein assay (BCA) according to the manufacturer’s instructions (Sigma, #B9643). The size and EV particle concentration were quantified by the NS300 Nanoparticle Tracking Analysis (NTA) system. Both measures were normalized by the initial volume of plasma per sample. The protein/particle ratio was determined as EV cargo (EVc).

**Mass spectrometry:** LC-MS/MS was used to characterize EV protein content. Peptides were analyzed by nano-LC-MS/MS using a Q-Exactive mass spectrometer coupled to an EASY-nLC 1000 liquid chromatography system (Thermo Fisher Scientific, Waltham, Massachusetts, United States) *via* the Nanospray Flex Ion Source.

**Statistics:** Differences in EV characteristics were tested with the linear mixed-effect model (“lme4” package) (11, 12). The EV sample correlation used the Spearman rank-order correlation (r) test; its strength was considered: r < 0.3, poor; 0.3 ≤ r <0.6, fair; 0.6 ≤ r <0.8, moderately strong, and 0.8 ≤ r, very strong (13). The variable association was tested through Pearson’s chi-squared test/Fisher’s exact test. Differently expressed EV proteins and protein expression correlations (*duplicateCorrelation* function) were analyzed using the “limma” package. The unadjusted hypergeometric p-value was used to compare functional protein enrichment between the groups of differentially expressed proteins. The *surv_cutpoint* function from the “survminer” package (14) was used to obtain the optimal cut-off point with the most significant relation to survival for EV characteristics (min. 0.25 observations/group). The multivariable logistic regression model for binary longitudinal data and Cox proportional hazards models used the “bild” (15) and “survival” (16) packages, respectively, with the inclusion/exclusion p-value criterion of 0.2 (17). The Wald test was used for parameter significance testing. “Survival” (16) and “survminer” (14) packages estimated the survival functions and computed the Kaplan–Meier survival curves. For the differences between survival estimates, the log-rank test was used. Unless otherwise stated, the significance is at the 5% level. Two-tailed p-value ≤ 0.05*, <0.01** and < 0.001*** were considered significant.. R software was used for analysis (18).

## 3 Results

### 3.1 Cohort Description

A total of 102 patients (MGUS=38, SMM=13, and MM=51) and 19 HDs were included. Baseline characteristics are summarized in **Table 1**. The cohort median follow-up time was 25.18 (95% CI: 21.0–31.13) months: 27.12 (95% CI: 19.82–31.20) months for MGUS, 23.54 (95% CI: 18.0–38.0 months for SMM, and 25.18 (95% CI: 20.8–35.4) months for MM patients. At the study inclusion, 21 patients were classified as MM-ND (before first-line treatment), 24 were classified as MM-NR, and 6 were classified as MM-R. According to the approved regimens, patients could have received a proteasome inhibitor (bortezomib, carfilzomib, or ixazomib), an immunomodulatory agent (lenalidomide or pomalidomide), or anti-CD38 monoclonal antibodies (daratumumab).

**Table 1:** Patient characteristics and distribution according to diagnostic category at study entrance.

Patient characteristics	HD	MGUS	SMM	MM
N subjects	n=19	n=38	n=13	n=51
Age range, y* N (%)				
<40	4 (21)	2 (5)	0 (0)	0 (0)
40 to <71	8 (42)	24 (63)	9 (70)	24 (47)
71 to <81	6 (32)	9 (24)	2 (15)	24 (47)
81 or higher	1 (5)	3 (8)	2 (15)	3 (6)
Median age, y*(min–max)	55 (30–82)	64 (37–87)	67 (43–83)	71 (43–86)
Sex, N (%)				
Female	9 (47)	15 (39)	9 (69)	20 (39)
Male	10 (53)	23 (61)	4 (31)	31 (61)
Risk score, N (%)	N/A	Low, 17 (45)	Low, 6 (46)	I, 17 (33)
		Low-Int., 12 (31)	Int., 5 (39)	II, 23 (45)
		High-Int., 9 (24)	High, 2 (15)	III, 11 (22)
		High, 0 (0)		

Risk score stratifications according to the diagnostic category: MGUS, monoclonal gammopathy of uncertain significance (9); SMM, smoldering multiple myeloma (19, 20); MM, multipl myeloma (9). N, number; HD, healthy donor; N/A, not applicable; y*, years; int, intermediate.

### 3.2 Circulating EV Characterization

**Figure 1:**
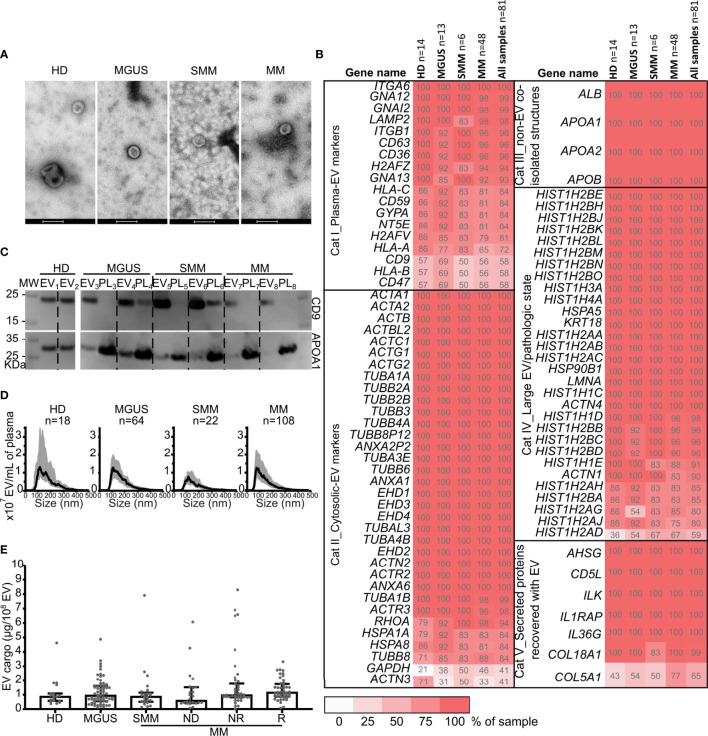
Characterization of extracellular vesicles from patient peripheral blood according to diagnostic category. **(A)** Transmission electron microscopy of circulating EV from representative samples from HD, MGUS, SMM, and MM samples. Scale bar, 200 nm. **(B)** Positivity for conventional protein markers identified by mass spectrometry across patients’ groups according to MISEV (3).The percentage of the samples expressing the specified protein is noted in each box. Dark red depicts a higher frequency. **(C)** Western blot analysis of CD9 and APOA-1 EV markers in EV samples and the source plasma (PL) sample. Two representative samples per group of diagnosis were tested. **(D)** Particle concentrations in plasma according to the size distribution from peripheral blood samples used in the present study (median and interquartile range). **(E)** EV cargo data distribution from peripheral blood samples according to the disease category at sample collection, HD (n=19 subjects, 19 samples), MGUS (n=38 patients, 67 samples), and MM (n=65 patients,137 samples): SMM (n=13 patients, 25 samples); ND (n=24 patients, 24 samples), NR, non-responder (n=32 patients, 42 samples), R, responder (n=27 patients, 46 samples). Using a linear mixed-effect model analysis, no significant differences between diagnostic categories (HD, MGUS, SMM, ND, NR, and R) were observed (p=0.39). HD, healthy donor; MGUS, monoclonal gammopathy of uncertain significance; SMM, smoldering multiple myeloma; NR, non-responder; R, responder, MM, multiple myeloma; EV, extracellular vesicle; PL, plasma; MW, molecular weight marker.

EV were isolated from 223 PB (circulating EV) and from 111 BM samples, and all were analyzed by NTA and BCA. Samples and EV characteristics according to MGUS, SMM, and MM (ND, NR, R) subgroups are described in **Figure S1** and **Table S1**. The EV morphology and size were confirmed by TEM (**Figure 1A**). The specificity of isolated EV was confirmed by the presence of several conventional EV markers (3) by MS (**Figure 1B**). The purity of EV was confirmed by Western blot in representative samples, where higher CD9 and lower APOA1 expressions were observed in EV samples compared to the unpurified plasma samples of origin (**Figure 1C**). The average modal size of EV was <150 nm in all groups (**Figure 1D**), showing a specific enrichment of the samples in small EV. Using a mixed-effect model, no significant differences in EVc, EV protein, or particle concentrations were found, either for PB or BM samples (**Figures 1E** and **S2A–F**) between MGUS, SMM, MM-ND, MM-NR, and MM-R subgroups. There were no significant associations between the EVc level and age or sex (Pearson’s chi-squared test, p-value=0.139 and 0.527, respectively).

### 3.3 EV Protein Content

#### 3.3.1 EV in PB as “Circulating” Counterpart of the BM

Direct comparisons were made between EV-PB and EV-BM from paired samples retrieved from the same patient. Among the 93 patients with paired PB and BM samples at study inclusion, a significant positive linear correlation was found in the EV protein concentration between PB and BM samples in MGUS (Spearman correlation r=0.63, p<0.001, n=37, **Figure S2G**) and MM patients (Spearman correlation r=0.77, p<0.001, n=56, **Figure S2H**). This means that a high protein concentration within circulating EV was associated with a high EV protein concentration in BM for both MGUS and MM patients, however, to a lesser extent in MGUS compared to MM.

**Figure 2:**
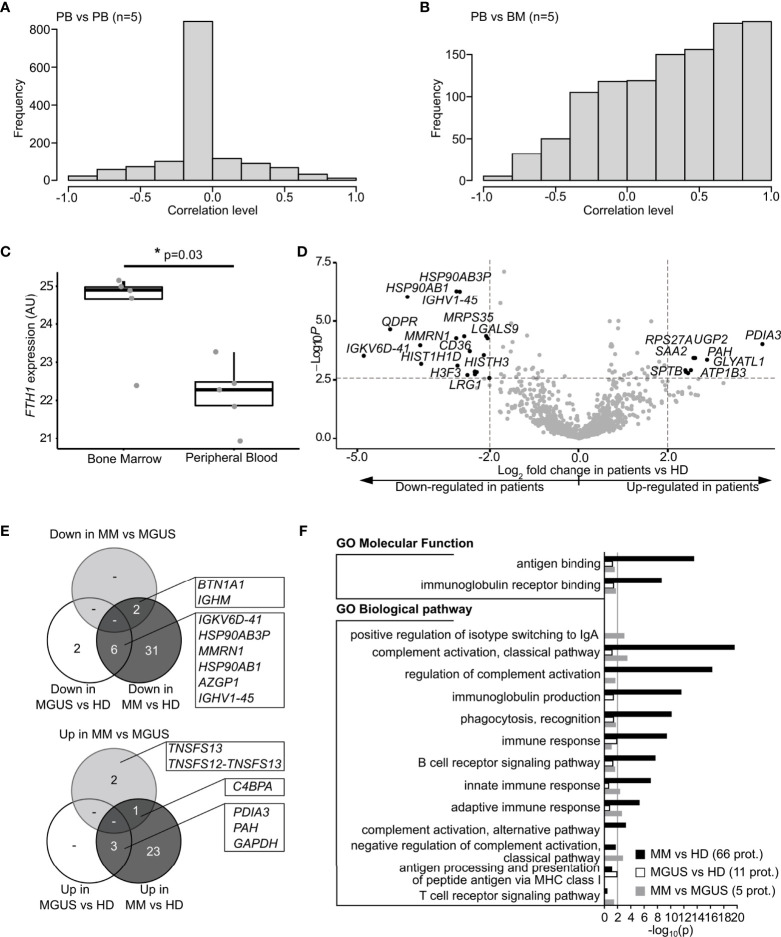
Characterization of proteins identified in EV from peripheral blood and bone marrow according to the diagnostic group. **(A)** Correlations between PB-matched samples from 5 pairs of patients (10 samples). Samples were matched considering the myeloma status at sample collection, existence of extramedullary disease, R-ISS status, number of previous treatment lines, and depth of response to treatment. The correlation for each protein is stored on as hyperbolic arctan (correlation). In this case, the correlations are equally distributed between positive and negative values but present a mode around 0. In the “limma” package, the consensus correlation is computed by discarding the most extreme outliers, averaging the remainder on the hyperbolic arctan scale, and then transforming back to a correlation. **(B)** Correlations between PB and BM samples collected from the same 5 patients at matched time points (10 samples). Correlation was computed as described in **(A)**. For this case, the correlations are mainly positive and have a mode around 0.8. **(C)** Boxplot of iBAQ values obtained for Ferritin Heavy Chain 1 (*FTH1*). Matched PB and BM samples from 5 patients were compared (10 samples). The LC-MS/MS data were quantified by three different computational methods to ensure a consistent computational analysis: spectral counting, iBAQ quantitation based on ion counts, and iBAQ quantification based on ion counts using the match between runs. For all three analyses, *FTH1* was significantly more abundant in BM samples, even after correction for multiple testing. **(D)** Volcano plot representation of differentially expressed proteins between all monoclonal gammopathy patients analyzed together and HD: differential expression of proteins above the horizontal line is significant with p-adjusted <0.05; differential expression of proteins outside the vertical dotted lines indicates a 2-logarithimical fold change (log_2_ FC) <-2 or >2. **(E)** Venn diagrams representing significant differentially expressed proteins of downregulated (top panel) and upregulated (bottom panel) proteins in peripheral blood-derived EV between patient subgroups (MM and MGUS) and HD. Proteins with overlap between the 3 comparisons (MM vs. MGUS, MGUS vs. HD, and MM vs. HD) are described in boxes. **(F)** Functional enrichment analysis of differentially expressed proteins using the FunRich software. Enrichment of immune-related molecular function and biological process was ranked for all deregulated proteins in MGUS and MM patient subgroups compared to healthy donors’ EV. PB, peripheral blood; R-ISS, revised international staging system; BM, bone marrow; AU, arbitrary unit; HD, healthy donor; MGUS, monoclonal gammopathy of uncertain significance; MM, multiple myeloma; EV, extracellular vesicle; prot, proteins.

Comparative EV protein profile analysis using LC-MS/MS results was performed in paired PB and BM samples from five patients (1 MGUS and 4 MM). An overall correlation of protein expression levels between the PB/BM pairs of 0.4 was found (**Figure 2B**). Then, the PB EV protein profile was compared to PB EV samples from other patients, matched for MM ND, R, and NR to understand the potential influence of the disease status in protein expression. Here, no correlation of protein expression levels between PB/PB-matched samples was observed (**Figure 2A**). This suggests a higher PB/BM proteomic expression similarity and supports the potential use of circulating EV as a personalized counterpart of the BM EV proteome. Interestedly, the major difference in proteomic expression between PB/BM-paired samples was in ferritin heavy chain 1 (*FTH1*), which was significantly more abundant in BM versus PB samples (T-test p-value=0.03, log_2_ FC=2.2, **Figure 2C**), corroborating the sample origin.

#### 3.3.2 EV Proteomic Expression in PB

EV proteomic expression was analyzed in a total of 51 patients with monoclonal gammopathies (MGs) including 13 MGUS, 5 SMM, and 33 MM.

First, a “disease”-blinded approach was performed by comparing EV proteomic expression in the PB of all the 51 MG patients together to HDs (n=14). Functional enrichment analysis using FunRich software (version3.1.3_March 2017) (21) against the Vesiclepedia database showed that 287 proteins were not annotated in this database. Interestingly, a higher enrichment in biological processes related to the immune response was observed in EV from all MG cohorts compared to the database. Within the 1,518 proteins identified, 82 were differently expressed in all MG patients compared to HDs (Benjamini–Hochberg-adjusted p-value <0.05) with 35 upregulated and 47 downregulated in patients. From these, the disulfide isomerase A3 precursor (*PDIA3*) required for protein folding in the endoplasmatic reticulum (22) had the highest positive fold change in MG patients, compared to HDs (**Figure 2D**). Comparisons between SMM and other disease status subgroups (MGUS, MM-ND, MM-NR, and MM-R) showed only the upregulation of collectin-11 (*COLEC11*) protein (log_2_ FC= 2.29) in patients responding to treatment, with no additional differences.

Considering this, we analyzed possible differences between HDs, MGUS, and MM (including SMM) and found that from the total of 5 proteins differentially expressed between MM and MGUS, Complement component 4 binding protein Alpha (*C4BPA*) protein was upregulated in MM patients (**Figure 2E**, bottom). C4bp is a regulator of complement activation that accelerates the decay of the classical pathway (23). Regarding the alternative pathway, Complement C3 (*C3*) and Complement factor H **(** *CFH*) were upregulated in MM vs. HDs, with a lower fold-change compared to the classical pathway. On the other hand, immunoglobulin heavy constant mu (*IGHM*) and butyrophilin subfamily 1 member A1 (*BTN1A1*) were downregulated in myeloma patients (**Figure 2E**, top). *BTN1A1* contains immunomodulatory functions as a B7 family member (24). The tumor necrosis factor ligand superfamily member 13 or APRIL (*TNFSF13*) and *TNFSF12-TNFSF13* were significantly upregulated in EV from MGUS patients when compared to myeloma patient EV. APRIL is usually secreted as a soluble molecule and binds with high affinity to the B-cell maturation antigen (*BCMA*), with higher serum levels in MM (25). Functional enrichment analysis was performed on differentially expressed proteins between groups (HD, MGUS, MM) focusing on molecular functions and biological processes related to immune functions as the most enriched categories. Differentially expressed proteins between HD, MGUS, and MM groups are strongly related to innate and adaptive immune responses. These enrichments were more pronounced when comparing MM vs. HDs, than MGUS vs. HDs, supporting a higher number of immune function alterations in MM patients (**Figure 2F**).

### 3.4 Circulating EV Cargo and Patient Outcomes

#### 3.4.1 High EVc in Myeloma Patients Is Related to Lower Survival

**Figure 3:**
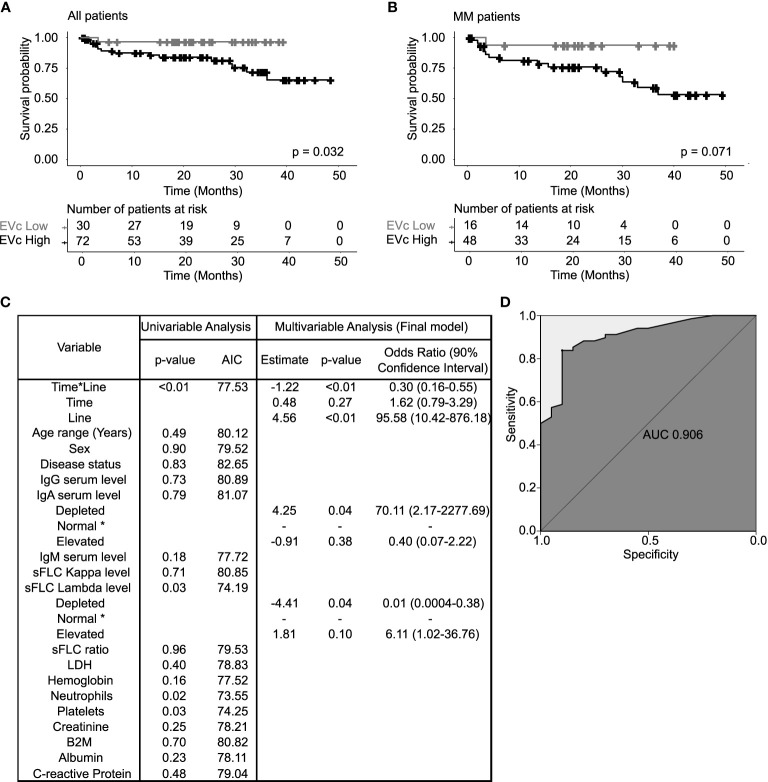
Extracellular vesicle cargo (EVc) characterization in terms of patients’ survival and longitudinal assessment. EVc, or protein-to-particle ratio, was defined by the ratio between the EV protein concentration and the EV particle concentration, for each EV sample. **(A)** Kaplan–Meier curves of patients’ overall survival (OS) according to the EVc level and diagnostic category. Applying the same method for optimal cut-off assessment as used for EV protein and particle concentration, MG patients (MGUS, SMM, and MM) with high EVc (>0.6 µg/10^8^ EV) had a statistically significant lower OS when compared to patients with low EVc (≤0.6 µg/10^8^ EV; log-rank test, p-value=0.032). **(B)** Comparing the specific EVc levels for MM (with SMM) patients, one can state that EVc High (>0.6 µg/10^8^ particles) patients always present a worse prognosis than EVc Low (≤0.6 µg/10^8^ particles) (considering a significance level of 10%: log-rank test, p-value=0.071). **(C)** Description of the variables tested to predict the odds of patients having a high EV cargo over time (SMM, MM-ND, MM-NR, and MM-R patients). Patient laboratory parameters obtained between 30 days before and 1 week after EV collection were used. *Time*—the chronological sequence of sample collection in each patient; *line*—the number of previous treatment lines. A variable of the interactions between *time* and *line* variables (time*line) was used to infer time in response (number of samples collected within the same line of treatment). The final model (multivariable analysis) resulted from a stepwise selection procedure, where variables were added one at a time and singularly tested in a univariable model. Wald test was used as a method for parameter significance testing. Model selection was performed through likelihood ratio test (LRT) computation, considering an inclusion/exclusion p-value set at 0.2 as described by Bendel and Afifi (17). IgA serum level, sFLC lambda level, and time*line were significant in the final model (p<0.1). **(D)** Final model goodness-of-fit graphical representation by the receiver operating characteristic (ROC) curve with an area under the curve (AUC) of 0.906. AIC (Akaike information criterion); * (reference class). sFLC, serum free light chain; B2M, β_2_microglobulin; LDH, lactate dehydrogenase.

The potential prognostic function of EV characteristics was investigated in the entire MG population with the intent of exploring them as new biomarkers independently of the disease category. These included the protein concentration, particle concentration, and protein/particle ratio (EVc). EV protein and particle concentrations had no impact in patient OS (**Figures S3A, B**, respectively). However, patients with EVc > 0.6 µg/10^8^ particles (high EVc) had a significantly shorter OS compared to patients with EVc ≤ 0.6 µg/10^8^ particles (low EVc), with survival probability at 25 months of 84% (95% CI: 0.75–0.94) vs. 97% (95% CI: 0.91–1), respectively (log-rank test, p-value=0.032, **Figure  3A**). Moreover, at 10% confidence level, the proposed cut-off point significantly differentiates MM patients with a better (low EVc) vs. poorer (high EVc) prognosis (**Figure 3B**, log-rank test, p-value=0.071). In detail, MM patients with high EVc presented a shorter probability of surviving beyond 25 months when compared to patients with low EVc (76% vs. 94%, respectively) with 90% CI equals to 0.66–0.88 and 0.84–1, respectively. Univariable and multivariable Cox proportional hazards models were developed to understand the role of EVc in the MM patient survival (**Table S2**). In addition to increased age, high β_2-_microglobulin, and MM-NR, patients with high EVc have a higher risk of death when compared to patients with low EVc (HR 12.23, p-value=0.028).

#### 3.4.2 High EVc in Myeloma Patients Can Be Predicted by Immunoparesis, High Serum Free Light Chain Level, and Shorter Time in Response

According to the results from the previous section, a multivariable longitudinal logistic regression model, estimating the odds of MM patients having a high EVc across the study time (for SMM, MM-ND, MM-NR, and MM-R) was built to determine if common myeloma-related blood parameters can explain the predictive value of EVc on MM OS (**Figure 3C**) in our cohort. Patient characteristics according to the EVc level are described in **Table S3**. IgA and sFLC lambda serum levels together with the time*line interaction were associated with high EVc, with an area under the ROC curve of 0.91 (**Figure 3D**). Patients with immunoparesis (IgA <50 mg/dl) had an increase of 70 times in the odds of high EVc compared to patients with normal IgA. Interestingly, among the 26 patients included in the model who presented IgA depletion, 22 were of the IgG subtype, suggesting that the immunoparesis of IgA was mostly associated to uninvolved Ig. Patients with elevated sFLC lambda (>27 mg/L) had an increase of 6 times in the odds of high EVc compared to patients with normal levels. Patients with low levels of sFLC lambda (<8.3 mg/L) presented an average reduction of 98% in the odds of having a high EVc compared to patients with normal levels. Patients with longer time in response showed an average decrease of 70% in the odds of having a high EVc compared to patients with a shorter response period. Furthermore, EV LC-MS/MS analysis was performed in a subgroup of 38 myeloma patients with high and low EVc (38 patients/51 samples) to explore the influence of patient serum IgA depletion and sFLC lambda elevation in the EV protein content. Interestingly, myeloma patients with IgA depletion had a significant downregulation of immunoglobulin heavy constant alpha 1 and 2 (*IGHA1* and *IGHA2*) when compared to patients with normal and elevated levels of IgA. Furthermore, patients with sFLC lambda elevation had an upregulation of proteins related to the immunoglobulin lambda constant (*IGLC1, IGLC2, IGLC3, and IGLC6*); immunoglobulin lambda-like polypeptides (*IGLL1* and *IGLL5*) and ferritin heavy chain 1 (*FTH1*), when compared to patients with the depletion of sFLC lambda.

## 4 Discussion

### 4.1 EV in a Real-World Study

To our knowledge, this is the first time that the EV protein content from an entire real-world MG patient cohort, prospectively followed in clinical practice for more than 2 years, is presented. The descriptive and comprehensive nature of our study generated a large set of real-world data from PB and BM EV derived from MG patients. In the 223 PB and 111 BM samples collected, the size and shape of the isolated particles were analyzed, and enrichment in EV markers was confirmed. Samples were tested for EV contaminants and were all coherent with purified-EV samples. The EV protein content from patient PB samples revealed a strong positive correlation with matched BM samples, as confirmed by proteomic expression. This is in line with the description of the BM proteome being more related to the blood proteome than to other tissues (26). However, in our study, BM samples were only collected from patients and therefore, this observation cannot be inferred to HDs. *FTH1*, the heavy chain of ferritin, a major intracellular iron storage protein, was found with higher expression in BM compared to PB samples, supporting the circulating EV protein content as liquid biopsies in MM. Considering this, we analyzed differences in PB EV-protein expression in a larger set of MG patients.

### 4.2 High EVc Level in Myeloma Patients Is Related to Poor Outcomes

In this work, we examined the extent to which EV characteristics, such as protein concentration, particle concentration, and the protein/particle ratio (EVc), could be a predictor of survival in patients with MG, regardless of the disease status allocation at study inclusion. Since the study of the EV characteristics in MG had not been conducted so far, there was no prior knowledge of their influence in combination with the disease status regarding the time to death. For that reason, we first investigated the optimal cut­off point better defining an MG patients’ prognosis by applying an outcome-oriented statistical method. We demonstrated for the first time that the EVc level is significantly associated with OS. We identified the value of 0.6 µg/10^8^ particles as the optimal cut-off above which high EVc myeloma patients had a significantly shorter OS compared to patients with low EVc (≤0.6 µg/10^8^ particles). As patients were included at any point in their disease history, the samples at study entry reflected the real-world heterogeneity of myeloma patients followed in clinical practice. Despite this cohort clinical diversity, circulating EVc alone was able to accurately identify patients with poor outcomes. This result was confirmed in a multivariable Cox regression model analysis accounting for age, β_2-_microglobulin, and disease status, ultimately indicating that myeloma patients with high EVc had 12 times increased risk of dying. Until now, the EVc has been typically described as quality control for EV purification (27) and, to our knowledge, this has not been associated with patient outcomes.

A multivariable logistic regression model fitted for longitudinal data was developed to determine whether common myeloma-related blood parameters could explain high EVc. Our model associated high EVc in MM patients with high sFLC lambda levels (>27 mg/L), immunoparesis (IgA <50 mg/dl), and shorter time in response. The utility of sFLC for the diagnosis, prognosis, and monitoring of MG is recognized in international guidelines (28). Extreme elevations of sFLC values and highly abnormal κ/λ ratios at the baseline are associated with refractory disease (29, 30) and are used to stratify SMM (19, 20). Our results confirmed the previously reported association of sFLC and EV (31–33). Interestingly, Di Noto et al. showed that endothelial and heart muscle cell lines reroute FLCs *via* EV (32) and that EV pre-treatment with anti-FLCs antibodies block MM EV uptake (31). Concerning IgA depletion, if immunoparesis was already associated with MM patient–reduced survival (34), its relation with circulating EV has not been described so far. Our results support EVc as a promising prognostic biomarker for MM patients, as it is associated with important prognostic features such as immunoparesis, sFLC, or the duration of the treatment response. To explore the potential biological link between high EVc and these immune alterations, LC-MS/MS was performed in circulating EV. The downregulation of *IGHA1/IGHA* confirmed IgA depletion in patients with immunoparesis. The upregulation of proteins related to Ig lambda production in patients with elevated sFLC lambda levels confirmed the increased presence of sFLC in these patients’ EV. EVc is an indirect and more affordable measure to infer EV protein load compared to mass spectrometry. After validation in larger and independent cohorts, EVc could be used as a first approach to predict patients with poor survival and select those at higher risk to be further analyzed by LC MS/MS.

### 4.3 Circulating EV as Source of New Myeloma Biomarkers

Eighty-two proteins were differentially expressed in MG patients compared to HDs. The functional analysis of differentially expressed proteins unraveled circulating EV as potential new tools for the dynamic interception of cellular communications in MM. Most downregulated proteins were involved in innate and adaptive immune responses, as proteins expressed in physiological conditions are lost in immune impaired myeloma patients (35, 36). EV are known to play important roles in immune phenomena (37), such as those from dendritic and B cells carrying major histocompatibility complex class (MHC), co-stimulatory, and adhesion molecules (38–40). In MM, the proteomic analyses of EV from MM cell lines and patient serum showed that MHC-I and β_2_-microglobulin were the most abundant enriched proteins (41).

Here, we describe a set of specific proteins from MG patients circulating EV. We propose *PDIA3* as a potential disease biomarker for MG, present since the MGUS stage. *PDIA3* codes the protein disulfide isomerase (PDI)–like family (22, 42). In cancer, PDI expression is upregulated in various tumors with poor outcomes (43). *PDIA3* is involved in the antigen presentation pathway of folding, assembly, and peptide loading of MHC-I (42). With the inhibition of PDI in the endoplasmic reticulum (ER), increasing ER stress could be a new strategy for MM treatment (44). A recent pan-PDI inhibitor enhanced the cytotoxic effects of proteasome inhibitors in MM models (45). Together with our results, circulating EV-derived *PDIA3* could be potentially used for PDI inhibition treatment monitoring. Additionally, we showed that immunomodulatory functions were associated to MG-specific disease stages. *BTN1A1* codes a protein related to the B7 family of costimulatory molecules and was significantly downregulated in myeloma patients, compared to HDs and MGUS. Conversely, myeloma patients presented a significant and consistent upregulation of *C4BPA*, a regulator of complement activation that accelerates the decay of the classical pathway. The sequestration of plasma regulators, such as *C4BPA*, by tumor cells has been shown as a mechanism to prevent complement-dependent cytotoxicity (23, 46). Complement activation is an important cause of inflammation involved in tumor progression (47). The expression of EV proteins such as *IGHM* and *C4BPA*, related to the classical pathway, suggest an alteration of this pathway in MGUS and to a greater extent in MM, compared to HDs. A proteomic analysis of MM patient serum samples reported the upregulation of complement activation proteins, notably C4B (48), which is also upregulated in our study. Surprisingly, we found a significant upregulation of APRIL, the natural ligand for BCMA, and TWE-PRIL proteins in MGUS patients’ EV compared to MM EV. This suggests a potential loss of expression in EV during disease progression, which may be related to APRIL higher increase in the serum of MM patients. Indeed, other studies have shown an increase of serum APRIL levels in patients with MM (49). In our study, only the EV protein content was analyzed, as opposed to the entire patient serum. Based on our results, one can hypothesize that “richer” EV-derived APRIL in MGUS patients can be shed off from EV upon disease progression to MM (with “poorer” EV-derived APRIL). This hypothetical release of APRIL in MM from EV to serum could be directly related to the increase of malignant plasma cells and microenvironment cells that overexpress APRIL receptors upon disease progression. If confirmed, this has the potential to be an early biomarker of disease evolution. Our findings show that EV-derived proteins (*PDIA3*, *BTN1A1*, APRIL) and complement-associated proteins could be further explored in myeloma, as biomarkers across disease natural history and as potential new drug targets that could be monitored by PB sampling. To identify tumor-specific proteins as previously done (50), we selected the proteins that were commonly differentially expressed in matched pairs of BM and PB of MM patients vs. the PB of HDs. A total of 228 upregulated and 266 downregulated proteins in MM vs. HDs, that are potentially tumor specific were identified. Among them, we could find *C4BPA*, *IGHM*, and *BTN1A1* that we previously mentioned as being specifically related to MM patients, suggesting that they could be directly produced by the tumor. However, due to the limited sample size, this needs to be confirmed.

Of note, preliminary results on a small number of patients suggest the presence of upregulated proteins in MM patients resistant to the bortezomib–lenalidomide–dexamethasone (VRD) regimen when compared to patients resistant to autologous stem cell transplant or to MM patients resistant to daratumumab-based regimens (data not shown). Among these, 17 proteins were constantly upregulated in patients resistant to VRD, including the proteasome subunit beta proteins (*PSM8* and *PSMB8*). In patients resistant to the daratumumab regimen the high-affinity Camp-specific and IBMX-insensitive 3’,5’-cyclic phosphodiesterase 8B (*PDE8B*) was also significantly upregulated when compared to patients resistant to VRD. Despite being preliminary, these observations suggest that EV proteins have the potential to be used as the biomarkers of drug resistance and that further investigation is worthy. In the literature, the replacement of *PSMB5* by *PSMB8* is described as increasing the cleavage capacity of the immunoproteasome peptides (51). It is also known that daratumumab treatment modulates the enzymatic activity of CD38 by reducing the adenosine levels and that *PDE8B* is a regulator of cyclic adenosine monophosphate. Therefore, the upregulation of *PDE8B* as a potential *via* the resistance to the immunomodulatory effect of daratumumab through adenosine reduction (52) is worthy of further study.

## 5 Conclusions

In this work, we thoroughly describe EV protein content from real-world MG patients. We found that the level of circulating EVc (protein/particle ratio, easily obtained by BCA and NTA) can be related to patients’ poorer outcomes including OS, high sFLC, immunoparesis, and shorter time in response. By mass spectrometry analysis, we also report new candidate biomarkers that can be associated with different disease stages. Altogether, our results corroborate the pursuit of EV as new liquid biopsies in myeloma and future validation in independent clinical settings is urged.

## Data Availability Statement

The original contributions presented in the study are publicly available. This data can be found here: https://www.ebi.ac.uk/pride/archive/projects/PXD024121.

## Ethics Statement

The studies involving human participants were reviewed and approved by Champalimaud Foundation and Centro Hospitalar de Lisboa Ocidental Ethics Committees, respectively and by the Portuguese National Committee for Data Protection. The patients/participants provided their written informed consent to participate in this study.

## Author Contributions

Conceptualization, BF, CJ, and BC-S. Methodology, BF, EC, CP, RM, and CJ. Formal analysis, BF, EC, CP, FB, RL, PL, MN, BC-S, and CJ. Investigation, BF, EC, FB, JC, RL, HB, AC, and RM. Resources, HB, RM, and AC. Data curation, EC, FB, PL, MN, and CJ. Writing—original draft preparation, BF, EC, CP, MN, PL, BC-S, and CJ. Writing—review and editing, BF, EC, BC-S, and C.J. Supervision, BF and CJ. Project administration, CJ. Funding acquisition, BF and CJ. All authors have read and agreed to the published version of the manuscript.

## Funding

This research was funded by the Champalimaud Foundation; by the Fundação para a Ciência e Tecnologia – FCT (Research Grant PTDC/MEC-HEM/30315/2017) and by Sociedade Portuguesa de Hematologia -SPH (Initiation to Investigation Grant 2018).

## Conflict of Interest

The authors declare that the research was conducted in the absence of any commercial or financial relationships that could be construed as a potential conflict of interest.

## Publisher’s Note

All claims expressed in this article are solely those of the authors and do not necessarily represent those of their affiliated organizations, or those of the publisher, the editors and the reviewers. Any product that may be evaluated in this article, or claim that may be made by its manufacturer, is not guaranteed or endorsed by the publisher.
