# Supplementary material for: Patient-Derived Extracellular Vesicles Proteins as New Biomarkers in Multiple Myeloma - A Real-World Study
Source: Front Oncol. 2022 Jun 21;12:860849. doi: 10.3389/fonc.2022.860849 (PMC9254863; doi:10.3389/fonc.2022.860849)
Supplement: Supplementary file 1 [file DataSheet_1.pdf]

Supplementary Material

1 Supplementary Data

Clinical study: The study was approved by the Ethics Committees of involved institutions and the Portuguese National Committee for Data Protection (study approval number: 11304/2016). Patients could enter the study at any time during disease natural history. All study participants (patients and healthy donors) with autoimmune diseases(1,2), heavy smoking(3,4) or other neoplasm were excluded. Demographics and clinical data were collected. All authors had access to primary unidentified clinical data. Risk scores for monoclonal gammopathy of uncertain significance (MGUS) performed according Rajkumar et al.(5) Healthy donors (HD) were adults (>18 years old) that after informed consent collected only one peripheral blood (PB) sample at study entrance for extracellular vesicles (EV) isolation. MGUS patients collected samples every 6 to 12 months in case of stable disease, and/or at progressive disease. Multiple myeloma (MM) patients collected samples before initiating treatment and/or at response evaluation. A total of 7 patients were excluded: 4 patients with autoimmune disease, 2 heavy smokers and 1 patient after exclusion of monoclonal gammopathy.

Sample preparation and EV purification, preanalytical and technical analytical details: Patients samples were collected after peripheral venous blood drawn into separate Ethylenediaminetetraacetic acid (EDTA) tubes. Tubes were stored at 4 ºC in the upright position until centrifugation (< 2 h) at 500 g for 10 min. Then, the collected supernatant was centrifuged at 3000 g for 20 min at 4ºC to eliminate cellular debris, apoptotic bodies and stored at -80 ºC. After thawing at room temperature, the amount of plasma used for EV isolation per sample was registered. An average of 4.94 ± 2.43 mL [0.58-15.35] and 2.42 ± 0.86 mL [0.85-6.00] of plasma were respectively used from PB and BM samples for EV purification. Samples were centrifuged at 12000 g for 20 min in polycarbonate tubes (Beckman/Coulter #082355645) to eliminate larger particles. Then, the supernatant was ultracentrifuged at 100000 g for 70 min in polycarbonate tubes (Beckman/Coulter #082355645), followed by pellet resuspension in 14 mL of filtered Phosphate-Buffered Saline (PBS, Corning, NY, United States). This sample was pipetted on top of 4 mL of sucrose cushion (D_2O containing 1.2 g of protease-free sucrose and 96 mg of Tris-base adjusted to pH 7.4) and ultracentrifuged at 100000 g for 70 min in polycarbonate tubes (Beckman/Coulter #082355631). Four milliliters of the bottom fraction were aspirated with a 18G needle and taken to a final volume of 20 mL with filtered PBS. Then the sample was ultracentrifuged at 100000 g for 18 h in polycarbonate tubes (Beckman/Coulter #082355631). The EV-containing pellet was resuspended in filtered PBS and stored at -80 ºC until EV characterization. EV aliquots were made to reduce the number of freezing-thawing cycles per sample. All solutions used (PBS and sucrose cushion) were sterile (0.22 µm membrane-filtered). Ultracentrifugations were performed at 10ºC with rotors 50.4Ti or 70Ti (Beckman-Coulter, California, United States).

EV markers by Western blot (WB): CD9 and APOA1 labeling were used as transmembrane positive EV biomarker and as purity biomarker(6), respectively. Purified EV and source plasma samples were diluted in Bolt LDS Sample buffer (Life technologies, Carlsbad, CA, USA). Five micrograms of protein were loaded in each well of a 4-12 %, Bis-Tris, 1.0 mm Bolt Mini Protein Gel (Life technologies). Electrophoresis was carried out at 180 V for 24 min in MES SDS Running Buffer (Thermo Fisher Scientific, Waltham, MA, USA). Separated proteins were electroblotted onto iBlot PVDF membrane using the iBlot 2 dry blotting system (Thermo Fisher Scientific) at 20 V for 1 min,

23 V for 4 min, and 25 V for 2 min. Membranes were rinsed 5 min in PBS and nonspecific sites were blocked for 1 h in SuperBlock (TBS) blocking buffer (Thermo Fisher Scientific) and 0.1 % TBS Tween-20 buffer. Membranes were incubated overnight at 4 °C in blocking buffer, with CD9 Antibody (Ts9, cat# 10626D, 1:1000, Invitrogen, Waltham, MA, USA) or ApoA1 Antibody (532, cat# MA5-14732, 1:1,500, Invitrogen). Membranes were washed 3x10 min in 0.1 % Tween-20 in PBS (PBS-T) and incubated in Goat anti-Mouse IgG (H+L) Secondary Antibody, HRP (cat# 32430, 1:2500, Thermo Fisher Scientific), diluted in blocking buffer for 1.5 h at room temperature. Membranes were washed 2x10 min in PBS-T and 5 min in PBS. Signal was revealed by a 5 min-incubation in SuperSignal West Femto Maximum Sensitivity Substrate (Thermo Fisher Scientific). Luminescent signal was acquired with the Amersham AI600 Imager (Cytiva, Buckinghamshire, UK). Spectra Multicolor Broad Range Protein Ladder (Thermo Fisher Scientific) was used as molecular weight marker. Each western blot analysis was repeated at least in two independent replicates.

EV characterization (EV protein and particle concentrations) technical analytical details: EV protein concentration was analyzed by colorimetric bicinchoninic acid (BCA) protein assay (Sigma-Aldrich, St. Louis, MO, USA). For that, 10 µL of each diluted sample were mixed to 200 µL of colorimetric bicinchoninic acid (BCA) reagent in a 96-well microplate. The plate was incubated in for 30 min at 37 °C and absorbance was measured at 562 nm and protein concentration was determined from a bovine serum album (BSA) standard curve. EV particle size and concentration was analyzed using NS300 Nanoparticle Tracking Analysis (NTA) system with red laser (638 nm) (NanoSight, Salisbury, UK). For this, samples were pre-diluted in filtered PBS to achieve a concentration within the range for optimal NTA analysis ($1x10^8$ to $5x10^8$ particles/mL, 20 to 40 particles/frame). Video acquisitions were performed using a camera level of 16 and a threshold between 4 and 6. Five videos of 30s were captured per sample. Analysis of particle concentration per mL and size distribution were performed with the NTA software v3.4.

Full description of proteomic analysis (Nano-LC-MSMS): For each sample, 20 µg of protein were prepared and run in duplicate. After elution through a LC column, proteins were incubated with sequencing-grade trypsin. Peptide samples (1 µg) were analysed in duplicates by nano-LC-MSMS (Dionex RSLCnano 3000) coupled to a Q-Exactive Orbitrap mass spectrometer (Thermo Scientific) applying MS and LC settings as virtually as previously described (PMID: 33330473), or by coupling the same LC system to an orbitrap Exploris 480 mass spectrometer (Thermo Scientific) applying following LC and MS settings; The samples (5 µl) were loaded onto a custom made fused capillary pre-column (2 cm length, 360 µm OD, 75 µm ID packed with ReproSil Pur C18 3 µm resin (Dr Maish, GmbH)) with a flow of 5 µl/min for 7 min. Trapped peptides were separated on a custom made fused capillary column (25 cm length, 360 µm OD, 75 µm ID, packed with ReporSil Pur C13 1.9 µm resin) using a linear gradient ranging from 89 % solution A (0.1% formic acid) to 32% B (80% acetonitrile in 0.1% formic acid) over 56 min followed by a linear gradient to 50% B over 8 min at a flow rate of 250 nL per minute. Mass spectra were acquired in positive ion mode applying automatic data–dependent switch between an Orbitrap survey MS scan in the mass range of 350–1200 m/z followed by peptide fragmentation applying a normalized collisional energy of 30% in a 2 second duty cycle. Target value in the Orbitrap for MS scan was 1,000,000 ions at a resolution of 60,000 at m/z 200 and 200,000 ions at a resolution of 15,000 at m/z 200 for MS/MS scans. Ion selection threshold was set to 20,000 counts. Selected sequenced ions were dynamically excluded for 30 s. Database search - The obtained data from the 282 LC-MS runs were searched using VEMS.(7)·(8) A standard human proteome database from UniProt (3AUP000005640) for Permutated protein sequences, where Arg and Lys were not permutated, were included in the database. Trypsin cleavage allowing a maximum of 4 missed cleavages was used. Carbamidomethyl cysteine was included as fixed modification. Methionine

oxidation and N-terminal protein acetylation was included as variable modifications. 10 ppm mass accuracy was specified for precursor ions and 0.01 m/z for fragment ions. The false discovery rate (FDR) for protein identification was set to 1% for peptide and protein identifications. No restriction was applied for minimal peptide length for VEMS search. Identified proteins were divided into evidence groups as defined by Matthiesen et al.(8) Peptide Sample Preparation - Protein solutions containing SDS and DTT were loaded onto filtering columns and washed exhaustively with 8M urea in HEPES buffer.(9) Proteins were reduced with DTT and alkylated with IAA. Protein digestion was performed by overnight digestion with trypsin sequencing grade (Promega). Quantitative analysis - Quantitative data obtained from VEMS results were analyzed in R statistical programming language. Quantitative data was extracted for 85 samples (170 LCMS runs) from participants selected in this current study. Intensity based absolute quantitation (IBAQ)(10) were preprocessed by removing common MS contaminants. Quantitative values from duplicate runs were averaged followed by log2(x + 1) transformation and quantile normalization.

Statistical analysis: Tested variables for the logistic regression longitudinal model: myeloma status (SMM, MM-ND, MM-R, MM-NR), age, sex, *time, line* and common myeloma-related blood analysis: total serum immunoglobulins (IgA, IgG and IgM), serum free light chains (sFLC), sFLC ratio, hemoglobin (HB), $\beta_2$microglobulin (β_2M), lactate dehydrogenase (LDH), platelets, neutrophils, serum creatinine, serum albumin and C-reactive protein (CRP). Patient laboratory parameters obtained between 30 days before to 1 week after EV collection were used. A stepwise algorithm with an inclusion/exclusion p-value criterion of 0.2 was performed as described by Bendel and Afifi(11). Wald test was used for parameter significance testing. LC-MS/MS data for PB/BM matched pairs was quantified by spectral counting; Intensity based absolute quantitation (iBAQ) based on ion counts; and iBAQ based on ion counts using match between runs. Correlations of protein expression between PB and (1) matched BM samples from the same patient and (2) PB samples from disease matched patients were performed using the *duplicateCorrelation* function implemented in "limma" package(12) (R/Bioconductor software). Differences in EV characteristics were analyzed using linear mixed-effect model . EV samples correlation was tested through Spearman rank-order correlation (r) test and its strength was considered as follows: $r < 0.3$, poor; $0.3 \leq r < 0.6$, fair; $0.6 \leq r < 0.8$, moderately strong and $0.8 \leq r$, very strong. The *surv_cutpoint* function from "survminer" package allowed the search for the optimal cut-off point with the most significant relation to survival for EV characteristics, able to discriminate patients with a better and worse prognosis (considering a minimum proportion of 0.25 observations/ group). "survival" and "survminer" packages were used to estimate survival functions and compute Kaplan-Meier survival curves. Differences between survival estimates for the defined groups was assessed through log-rank test. Multivariable Cox proportional hazards model was developed to model the association between specific demographic and laboratory variables with OS in MM patients with complete laboratory information.

Supplemental References

1. Lam KCK, Lam MKN, Chim CS, Chan GCF, Li JCB. The functional role of surface molecules on extracellular vesicles in cancer, autoimmune diseases, and coagulopathy. J Leukoc Biol [Internet]. John Wiley & Sons, Ltd; 2020 Nov 1;108(5):1565–73. Available from: https://doi.org/10.1002/JLB.3MR0420-067R
2. Nielsen CT, Østergaard O, Stener L, Iversen L V, Truedsson L, Gullstrand B, et al. Increased IgG on cell-derived plasma microparticles in systemic lupus erythematosus is associated with autoantibodies and complement activation. Arthritis Rheum [Internet]. John Wiley & Sons, Ltd; 2012 Apr 1;64(4):1227–36. Available from: https://doi.org/10.1002/art.34381
3. Li M, Yu D, Jon Williams K, Liu ML. Tobacco smoke induces the generation of procoagulant

microvesicles from human monocytes/macrophages. Arterioscler Thromb Vasc Biol. NIH Public Access; 2010 Sep;30(9):1818–24.

4. Singh KP, Maremanda KP, Li D, Rahman I. Exosomal microRNAs are novel circulating biomarkers in cigarette, waterpipe smokers, E-cigarette users and dual smokers. BMC Med Genomics. BioMed Central Ltd; 2020 Sep;13(1).
5. Rajkumar SV, Kyle RA, Therneau TM, Melton LJ, Bradwell AR, Clark RJ, et al. Serum free light chain ratio is an independent risk factor for progression in monoclonal gammopathy of undetermined significance. Blood. Blood; 2005 Aug;106(3):812–7.
6. Théry C, Witwer KW, Aikawa E, Alcaraz MJ, Anderson JD, Andriantsitohaina R, et al. Minimal information for studies of extracellular vesicles 2018 (MISEV2018): a position statement of the International Society for Extracellular Vesicles and update of the MISEV2014 guidelines. J Extracell Vesicles. Taylor and Francis Ltd.; 2018 Jan;7(1).
7. Carvalho AS, Ribeiro H, Voabil P, Penque D, Jensen ON, Molina H, et al. Global Mass Spectrometry and Transcriptomics Array Based Drug Profiling Provides Novel Insight into Glucosamine Induced Endoplasmic Reticulum Stress*. Mol Cell Proteomics [Internet]. 2014;13(12):3294–307. Available from: https://www.sciencedirect.com/science/article/pii/S1535947620337580
8. Matthiesen R, Prieto G, Amorim A, Aloria K, Fullaondo A, Carvalho AS, et al. SIR: Deterministic protein inference from peptides assigned to MS data. J Proteomics [Internet]. 2012;75(13):4176–83. Available from: https://www.sciencedirect.com/science/article/pii/S1874391912002989
9. Wiśniewski JR, Zougman A, Nagaraj N, Mann M. Universal sample preparation method for proteome analysis. Nat Methods [Internet]. 2009;6(5):359–62. Available from: https://doi.org/10.1038/nmeth.1322
10. Schwanhäusser B, Busse D, Li N, Dittmar G, Schuchhardt J, Wolf J, et al. Global quantification of mammalian gene expression control. Nature [Internet]. 2011;473(7347):337–42. Available from: https://doi.org/10.1038/nature10098
11. Bendel RB, Afifi AA. Comparison of stopping rules in forward “stepwise” regression. J Am Stat Assoc. Taylor & Francis Group ; 1977;72(357):46–53.
12. Ritchie ME, Phipson B, Wu D, Hu Y, Law CW, Shi W, et al. limma powers differential expression analyses for RNA-sequencing and microarray studies. Nucleic Acids Res [Internet]. 2015 Apr 20;43(7):e47–e47. Available from: https://doi.org/10.1093/nar/gkv007

2 Supplementary Figures and Tables

2.1 Supplementary Figures

Figure S1

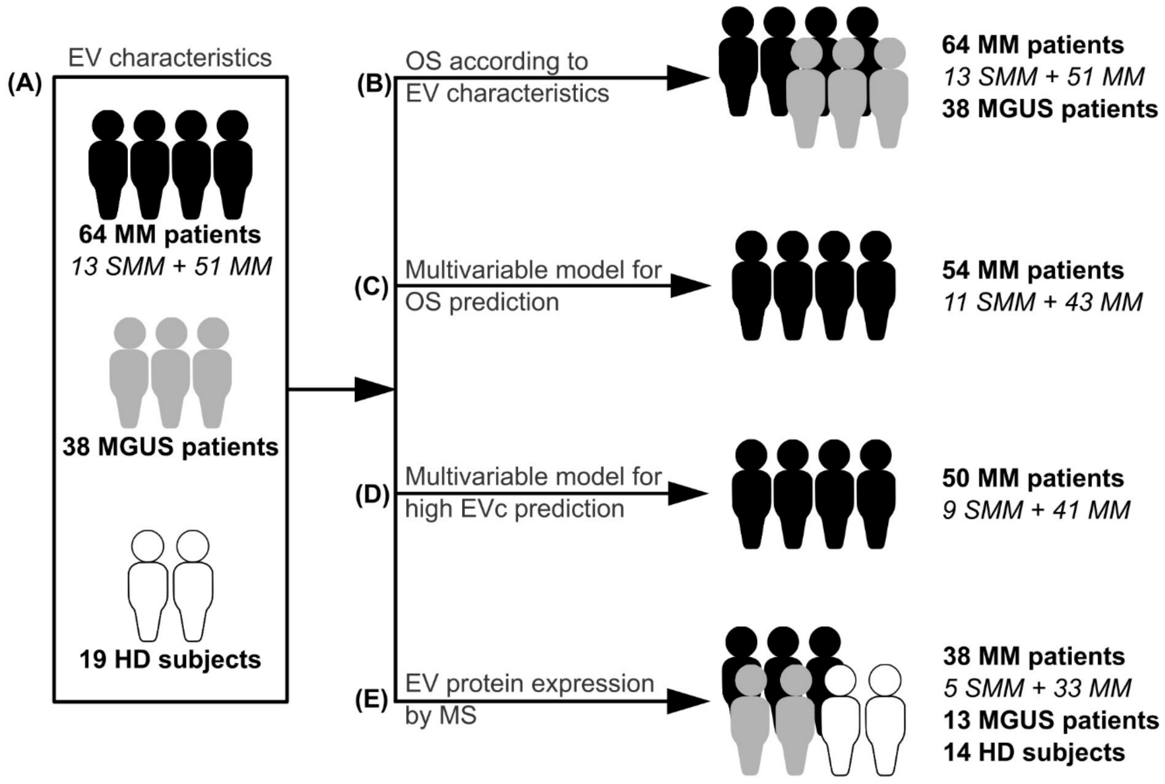

(F)

	HD	MGUS	MM			
			SMM	ND	NR	R
**Analysis**	**Peripheral Blood samples**					
**(A)**	19	67	25	24	42	46
**(B)**	-	38	13	21	24	6
**(C)**	-	-	11	19	21	3
**(D)**	-	-	15	16	29	28
**(E)**	14	13	6	8	16	22
**Analysis**	**Bone marrow samples**					
**(A)**		37	13	24	22	15
**(B)**		-	-	-	-	-
**(C)**	-	-	-	-	-	-
**(D)**		-	-	-	-	-
**(E)**		1	-	1	2	1

Supplemental Figure S1. Graphical representation of number of patients and samples distribution by diagnostic category and analyses. A total of 102 patients (204 PB and 111 BM samples) and 19 healthy donors (19 samples) were included in the study and analyzed for EV characteristics (**A**). For overall survival analyses patient samples at study entrance were used (**B**); for multivariable analyses, samples from patients with complete information were used according to each model (**C**, **D**); for mass spectrometry (MS) analysis, patient's samples were used according to sample viability and total protein content (**E**). Distribution of samples within each analysis according to patient diagnosis group (**F**). HD, healthy donor; MGUS, monoclonal gammopathy of uncertain significance; SMM, smoldering multiple myeloma; MM, multiple myeloma; ND, newly diagnosed; NR, non-responder; R, responder; EV, extracellular vesicle; PB, peripheral blood; BM, bone marrow; OS, overall survival; EVc, extracellular vesicle cargo.

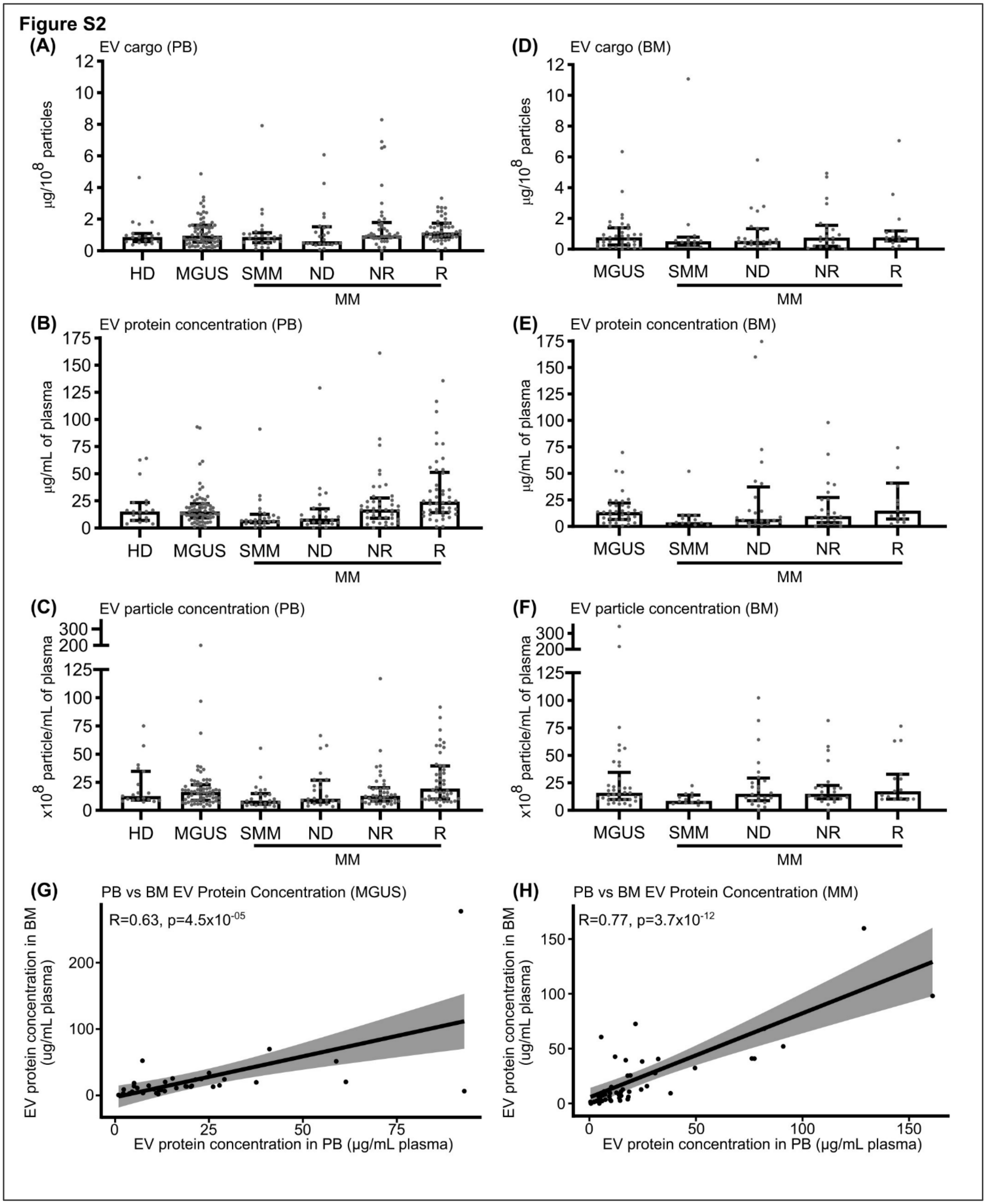

Supplemental Figure S2. EV cargo, protein and particle concentrations of peripheral blood (A-C) and bone marrow samples (D-F) according to diagnostic category. Using a linear mixed-effect model analysis, no significant differences between diagnostic categories were observed in PB and BM samples for EV cargo (**A and D,** p=0.39 and 0.91, respectively), for EV protein concentration (**B and**

E, p=0.11 and 0.31, respectively) nor for EV particle concentration (**C and F,** p=0.14 and 0.39, respectively). HD (n=19 subjects, 19 PB samples), MGUS (n=38 patients, 67 PB and 37 BM samples), MM (n=64 patients,137 PB and 74 BM samples): SMM (n=13 patients, 25 PB and 13 BM samples); ND, newly diagnosed (n=24 patients, 24 PB and 24 BM samples); NR, non-responders (n=32 patients, 42 PB and 22 BM samples); R, responders (n=27 patients, 46 PB and 15 BM samples). A significant positive linear correlation between BM and PB paired samples (from the same patient) at study inclusion was observed for MGUS patients (**G**) (Spearman correlation r=0.63, p<0.001, n=37) as well as for MM (including SMM) patients (**H**) (Spearman correlation r=0.77, p<0.001, n=56). HD, healthy donor; MGUS, monoclonal gammopathy of uncertain significance; SMM, smoldering multiple myeloma; MM, multiple myeloma; EV, extracellular vesicle.

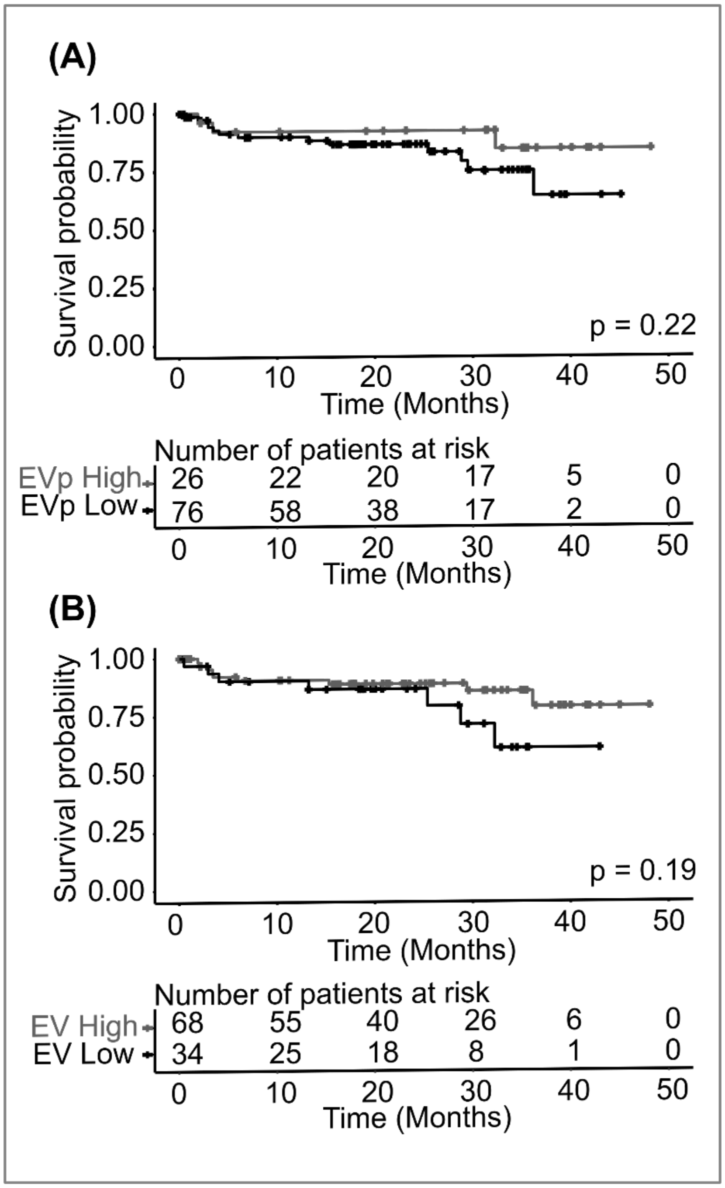

Supplemental Figure S3. Kaplan-Meier curves of patient overall survival according to extracellular vesicles (EV) characteristics. Overall survival (OS) of all patients according to the level of (A) EV protein concentration (EVp) and (B) EV particle concentration in patient peripheral blood. EV prognostic impact was analyzed by stratifying EV characteristics into two groups (high and low) based on their optimal cut-off point, as the most significant relation with survival. Stratifying each EV characteristic based on their optimal cut-off, no significant differences were observed between the levels of EV protein (≤ or >22.95 µg/mL; Log-rank test, p-value=0.225) or EV particle (≤ or >8.53 x10^8particles/mL; Log-rank test, p-value=0.19) concentrations regarding OS.

2.2 Supplementary Tables

Supplemental Table S1. EV characteristics of PB and BM samples prospectively collected and stratified according to diagnostic category. Median number of samples and volume of collected plasma per patient for each diagnostic category is indicated. EV size and particle concentration was analyzed by nanoparticle tracking analysis (NTA) system and EV protein concentration by bicinchoninic acid assay (BCA) protein assay. HD, healthy donor; MGUS, monoclonal gammopathy of uncertain significance; MM, multiple myeloma; EV, extracellular vesicle; N, number; PB, peripheral blood; BM, bone marrow.

**Samples characteristics**	**HD**	**MGUS**	**SMM**	**MM**
N samples				
PB	19	67	25	112
BM	-	37	13	61
N samples/patient Median (min-max)				
PB	1 (1-1)	2 (1-3)	2 (1-4)	1 (1-5)
BM	-	1 (1-1)	1 (1-2)	1 (1-3)
Collected plasma, mL Median (min-max)				
PB	6.35 (1.80-9.60)	3.59 (1.65-9.00)	4.50 (2.52-12.11)	4.03 (1.85-15.35)
BM	-	2.17 (0.93-5.0)	2.18 (1.91-4.95)	2.36 (0-85-6.0)
EV modal size, nm Median (min-max)				
PB	116.0 (98.0-154.70)	122.0 (88.0-252.0)	118.0 (88.0-150.0)	108.50 (77.0-194.0)
BM	-	131.0 (102.0-155.0)	129 (109-145.0)	125 (72.50-156.90)
Total EV particle concentration in plasma, 10^8EV/mL Median (min-max)				
PB	12.65 (7.44-74.99)	16.27 (3.51-200.72)	8.74 (2.72-55.28)	15.19 (3.37-116.95)
BM	-	16.14 (4.60-341.94)	8.66 (4.65-22.46)	15.90 (0-102.32)
EV protein concentration, µg/mL Median (min-max)				
PB	15.13 (3.80-64.04)	15.31 (0.92-92.96)	7.34 (0.94-90.97)	17.48 (0.04-161.05)
BM	-	13.40 (0-277.52)	3.81 (1.51-52.00)	9.77 (0-448.64)
EVc, µg/10^8EV Median (min-max)				
PB	0.88 (0.38-4.63)	0.95 (0.12-4.88)	0.88 (0.13-7.92)	1.04 (0.001-8.30)
BM	-	0.77 (0-6.34)	0.52 (0.15-11.07)	0.68 (0-7.06)

Supplemental Table S2. Multivariable Cox regression model. Description of the variables used to predict the risk of death for multiple myeloma (MM patients at baseline. The final model (multivariable analysis) resulted from a stepwise selection procedure, where variables were firstly added one at a time and tested singularly in a univariable model. Wald test was used as a method for parameter significance testing. Model selection was performed through likelihood ratio tests (LRT) computation considering an inclusion/exclusion p-value set at 0.2. Age, β2-microglobulin, disease status and EVc were significant in the final model (p≤0.1). *Reference class. LDH, lactate dehydrogenase; SMM, smoldering multiple myeloma; ND, newly diagnosed; R, responder; NR, non-responder; EVc, extracellular vesicle cargo; NA, not achieved.

**Variable**	**Univariable Analysis**	**Multivariable Analysis (final model)**		
	**p-value LRT**	**Coefficient**	**p-value**	**Hazard Ratio (90% Confidence Interval)**
LDH	0.90			
β_2 microglobulin	0.20			
Low		-0.088	0.908	0.916 (0.265-3.167)
Normal *		-	-	-
Elevated		1.911	0.049	6.763 (1.370-33.376)
Albumin	0.20			
Sex	0.70			
Age	0.50			
40-70 *		-	-	-
71-80		-2.259	0.006	0.105 (0.027-0.399)
>=81		2.178	0.052	8.828 (1.395-55.886)
Disease Status	0.02			
SMM+MM-ND *		-	-	-
MM-R		-20.340	0.999	1.467e-9 (0-NA)
MM-NR		2.258	0.005	9.564 (2.580-35.454)
EVc	0.04			
Low *		-	-	-
High		2.504	0.028	12.230 (1.876-79.788)
Lines	0.03			

Supplemental Table S3. Multivariable longitudinal logistic regression model patient' samples characteristics according to extracellular vesicles cargo (EVc) category (high or low) in peripheral blood. Median quantification number of sample and volume of collected plasma per patient is indicated. Q1, 1st quartile; Q3, 3rd quartile. N, number; y*, years; MM, multiple myeloma; SMM, smoldering multiple myeloma; ND, newly diagnosed; IgA, IgG and IgM, total serum immunoglobulins; sFLC, serum free light chains; HB, hemoglobin; β_2M, $\beta_2$microglobulin; LDH, lactate dehydrogenase; CRP, C-reactive protein.

EV cargo category	Low: ≤ 0.6	High: > 0.6
N samples	20	68
Median age, y* (min-max)	66.6 (42.5-84.8)	73.5 (42.8-86.2)
Age range, y* N (%)<br>40 to < 71 / 71 to < 81 / ≥ 81	12 (60) / 7 (35) / 1 (5)	26 (38) / 37 (54) / 5 (8)
Sex, N (%)<br>Female / Male	10 (50) / 10 (50)	28 (41) / 40 (59)
MM disease status, N (%)<br>SMM<br>MM-ND<br>MM-R / MM-NR	 6 (30)<br>9 (45)<br>2 (10) / 3 (15)	 9 (13)<br>7 (11)<br>26 (38) / 26 (38)
M protein isotype, N (%)<br>IgG / IgA / Other	13 (65) / 5 (25) / 2 (10)	46 (68) / 12 (17) / 10 (15)
Median IgG serum level, mg/dL (Q1-Q3)<br>IgG serum level category, N (%)<br>Depleted / Normal [600 – 1500]/ Elevated	1478.5 (579.7-3709.5)<br><br>6 (30) / 4 (20) / 10 (50)	794 (477.8-1774.8)<br><br>23 (34) / 26 (38) / 19 (28)
Median IgA serum level, mg/dL (Q1-Q3)<br>IgA serum level category, N (%)<br>Depleted / Normal [50 – 400]/ Elevated	75.5 (21.8-341.5)<br><br>7 (35) / 8 (40) / 5 (25)	61.5 (15-155.5)<br><br>33 (49) / 26 (38) / 9 (13)
Median IgM serum level, mg/dL (Q1-Q3)<br>IgM serum level category, N (%)<br>Depleted / Normal [50 – 300]/ Elevated	23 (10.5-50.0)<br><br>15 (75) / 5 (25) / 0 (0)	21.5 (14.8-49.8)<br><br>51 (75) / 17 (25) / 0 (0)
Involved sFLC, N (%)<br>Kappa / Lambda	15 (75) / 5 (25)	39 (57) / 29 (43)
Median sFLC Kappa level, mg/L (Q1-Q3)<br>sFLC Kappa level category, N (%)<br>Depleted / Normal [6.7 – 22.4] / Elevated	24.9 (14.7-354.0)<br><br>1 (5) / 8 (40) / 11 (55)	17.4 (10.3-51.1)<br><br>10 (15) / 30 (44) / 28 (41)
Median sFLC Lambda level, mg/L (Q1-Q3)<br>sFLC Lambda level category, N (%)<br>Depleted / Normal [8.3 – 27.0] / Elevated	7.8 (2.8-11.6)<br><br>11 (55) / 7 (35) / 2 (10)	14.0 (6.7-65.1)<br><br>22 (32) / 24 (36) / 22 (32)
Median sFLC ratio, (Q1-Q3)<br>sFLC ratio category, N (%)<br>Normal [0.26 - 1.65] / Abnormal <0.26 or >1.5	2.9 (1.2-52.2)<br><br>7 (35) / 13 (65)	1.2 (0.2-6.4)<br><br>23 (34) / 45 (66)

EV cargo category (continuation)	Low: ≤ 0.6	High: > 0.6
Median LDH level, UI/L (Q1-Q3)<br>LDH level category, N (%)<br>Normal ≤ 246 / Elevated > 246	201.0 (158.3-332.3)<br><br>13 (65) / 7 (35)	248.0 (178.8-376.0)<br><br>34 (50) / 34 (50)
Median HB level, g/dL (Q1-Q3)<br>HB level category, N (%)<br>Anemia < 10 / No anemia ≥ 10	11.4 (10.4-13.2)<br><br>1 (5) / 19 (95)	11.7 (10.4-12.8)<br><br>12 (18) / 56 (82)
Median neutrophils level, /mm^3 (Q1-Q3)<br>Neutrophils level category, N (%)<br>Low / Normal [2.5 - 7.5] / High	3.4 (3.0-4.7)<br><br>3 (15) / 17 (85) / 0 (0)	3.0 (2.1-4.5)<br><br>25 (37) / 35 (51) / 8 (12)
Median platelet level x10^3/mm^3 (Q1-Q3)<br>Platelet level category, N (%)<br>Low / Normal [150 - 400]/ High	256.0 (200.3-288.3)<br><br>1 (5) / 17 (85) / 2 (10)	170.0 (125.8-212.0)<br><br>29 (43) / 37 (54) / 2 (3)
Median calcium level mg/dL (Q1-Q3)<br>Calcium level category, N (%)<br>Normal < 11 / Hypercalcemia ≥ 11	9.5 (9.3-9.9)<br><br>20 (100) / 0 (0)	9.2 (8.8-9.5)<br><br>67 (99) / 1 (1)
Median creatinine level mg/dL (Q1-Q3)<br>Creatinine level category, N (%)<br>Normal ≤ 2 / Renal Insufficiency > 2	0.9 (0.7-1.2)<br><br>19 (95) / 1 (5)	0.9 (0.8-1.4)<br><br>61 (90) / 7 (10)
Median β_2M, mg/L (Q1-Q3)<br>β_2M level category, N (%)<br>Low / Normal [3.5 – 5.5] / High	2.9 (1.7-3.7)<br><br>14 (70) / 3 (15) / 3 (15)	3.2 (2.1-4.4)<br><br>40 (58) / 14 (21) / 14 (21)
Median albumin level g/dL (Q1-Q3)<br>Albumin level category, N (%)<br>Low ≤ 3.5 / Elevated > 3.5	4.1 (3.4-4.2)<br><br>6 (30) / 14 (70)	3.5 (3.3-3.9)<br><br>37 (54) / 31 (46)
Median CRP level, mg/dL (Q1-Q3)<br>CRP level category, N (%)<br>Normal ≤ 0.5 / Elevated > 0.5	0.2 (0.1-1.1)<br><br>13 (65) / 7 (35)	0.4 (0.1-1.1)<br><br>35 (51) / 33 (49)

Supplemental Table S3 (continuation)
